# Safety and efficacy of intravenous thrombolytic therapy in the extended window up to 24 hours: A systematic review and meta‐analysis

**DOI:** 10.1002/acn3.52239

**Published:** 2024-10-29

**Authors:** Omar M. Al‐Janabi, Seyed Behnam Jazayeri, Michelle A. Toruno, Yamama M. Mahmood, Sherief Ghozy, Shadi Yaghi, Alejandro A. Rabinstein, David F. Kallmes

**Affiliations:** ^1^ Department of Neurology Baptist Health Lexington Kentucky USA; ^2^ Sina Trauma & Surgery Research Center Tehran University of Medical Sciences Tehran Iran; ^3^ Department of Radiology Mayo Clinic Rochester Minnesota USA; ^4^ Centeral Pharmacy University of Kentucky Healthcare Lexington Kentucky USA; ^5^ Department of Neurologic Surgery Mayo Clinic Rochester Minnesota USA; ^6^ Department of Neurology Brown University Providence Rhode Island USA; ^7^ Department of Neurology Mayo Clinic Rochester Minnesota USA

## Abstract

**Objective:**

About 25% of patients with acute ischemic stroke (AIS) present within the intravenous thrombolytic (IVT) therapeutic window of <4.5 h. This study is to elucidate the safety and efficacy of IVT in the extended therapeutic window (ETW) in patients with AIS.

**Methods:**

Using PRISMA guidelines, a systematic review was conducted using PubMed, Embase, and Scopus. A rigorous risk of bias assessment was conducted using the RoB2 tool. Rates of excellent and good functional outcome (mRS 0–1 and mRS 0–2) at 90 days, symptomatic intracranial hemorrhage (sICH), and mortality at 90 days were pooled using generalized linear mixed model and compared with controls. Meta‐analyses were conducted employing random‐effect models with risk ratio (RR) and 95% confidence intervals (CIs). Subgroup analysis was performed to assess the effect of imaging modalities used for patient selection.

**Results:**

Eight randomized controlled trials (*n* = 2221, 59% male) were included. At 90 days IVT showed higher rates of functional recovery: mRS 0–1: RR 1.21 95% CI 1.1–1.34, *p* < 0.001, and mRS 0–2: RR 1.11 95% CI 1.03–1.18, *p* = 0.004. Rate of mortality at 90 day was not different between groups: RR 1.17 95% CI 0.93–1.48, *p* = 0.17. However, the rate of sICH was higher among IVT group: RR 2.93 95% CI 1.53–5.6, *p* = 0.001. Subgroup analysis showed higher mRS 0–1 among patients who were selected based on perfusion imaging (*p* < 0.05).

**Interpretation:**

The use of IVT in AIS in ETW is beneficial especially with the use of perfusion imaging for patients' selection.

## Introduction

Current guidelines recommend using intravenous thrombolysis (IVT) agents for patients with acute ischemic stroke (AIS) within 4.5 h of symptom onset. However, only about 5–25% of patients are eligible for this treatment due to the narrow therapeutic window.^1^ One reason for exclusion is patients with an unknown onset of symptoms, which occurs in cases of wake‐up or unwitnessed stroke where the symptom onset time cannot be estimated due to aphasia, impaired consciousness, or cognitive impairment.^2^ Several clinical trials have been conducted to extend the treatment window for patients with the potential to salvage penumbral brain tissue using imaging‐based selection, in addition to clinical criteria. For instance, the WAKE‐UP trial showed a significantly better functional outcome in patients with an unknown time of onset who were treated with intravenous alteplase (ALT) and MRI guidance.^3^ Conversely, the TIMELESS trial found no benefit in functional outcomes when tenecteplase (TNK) was administered 4.5 to 24 h after symptom onset based on a favorable perfusion‐imaging profile.^4^ It remains crucial to strengthen the evidence from previous research studies to improve the therapeutic management in this group of patients. Therefore, we conducted a systematic review and meta‐analysis of randomized controlled trials (RCT) evaluating the safety and efficacy of intravenous thrombolytics for AIS treatment in the extended window of more than 4.5 h after last known well (LKW), including subgroup analysis by thrombolytic type in extended therapeutic window (ETW).

## Methods

### Search strategy

PubMed, Embase, and Scopus were searched covering records from January 2000 to July 2024. We employed various combinations of keywords and Medical Subject Headings (MeSH) terms based on each database's requirements, including terms related to ischemic stroke, thrombolysis, thrombolytic agents and beyond 4.5 h. Reports were included without language, country, or date restrictions. The search strategy for Embase is presented in Supporting Information. Additionally, we manually reviewed references from articles that passed full‐text screening and conducted a thorough search of each included article's citations on Google Scholar to identify any relevant records.

### Eligibility criteria

Only randomized clinical trials (RCTs) evaluating the safety and efficacy of intravenous thrombolysis (IVT) with ALT and TNK in an ETW (after 4.5 h from stroke onset) were considered eligible.

Patient population included those with AIS who received thrombolytics in the ETW (Studies that reported mechanical thrombectomy in addition to IVT were also included). Comparison was made with the control group who did not receive thrombolytics in the ETW. Finally, outcome measures were functional outcomes, symptomatic intracranial hemorrhage (sICH), and mortality.

The exclusion criteria were receiving IVT below 4.5 h since wake up or LKW time, non‐RCT design, and nonhuman studies.

### Selection process

Three authors (O.M.A, S.B.J, and Y.M.M) independently assessed titles and abstracts based on predetermined criteria. In case of disagreement, a senior author facilitated consensus. The same three authors independently evaluated full‐text abstracts meeting inclusion criteria and reviewed references from systematic reviews. Additionally, one author (O.M.A) conducted citation checks for the articles included.

### Data extraction

An Excel‐based data extraction sheet was developed by one author (S.B.J), including study characteristics, baseline patient data, and relevant outcomes. Subsequently, two authors (S.B.J and O.M.A) independently performed data extraction. Any discrepancies were resolved through discussion and consensus.

### Quality appraisal

Two reviewers (S.B.J and O.M.A) assessed the quality of studies independently. We used the risk of bias assessment tool 2 (RoB2) of Cochrane to assess the quality of studies. Any disagreement between reviewers was resolved through consensus methods.

### Statistical analysis

Based on data availability, we performed meta‐analysis on four outcomes: (1) Excellent functional recovery at 90 days defined as modified Rankin Scale (mRS) scores of 0–1, (2) Good functional outcome defined as mRS 0–2, (3) sICH, and (4) mortality at 90 days. Given the varying criteria used to define sICH in different trials, we opted for the criteria that require an increase of at least 4 points in the NIHSS score, along with imaging evidence of ICH.

All analyses were conducted using R software version 4.3.2 (R Project for Statistical Computing) metafor package version 4.6–0. Generalized linear mixed model was used to calculate risk ratio (RR) and their corresponding 95% confidence intervals (CI) using random‐effects models.^5^ We used metaprop package to calculate proportional percentages and their 95% CIs for each outcome. Heterogeneity was assessed using Q statistic and the *I* ^2^ test, in which *I* ^2^ greater than 50% or *p* < 0.05 were considered significant. In the case of significant heterogeneity, a sensitivity analysis was performed with removal of outlier studies to bring the heterogeneity to an insignificant level. Outlier studies were identified using the method previously described by Viechtbauer and Cheung.^6^ We performed three subgroup analyses (1) based on the thrombolytic agent (ALT vs TNK) (2) The method of patient selection based on advanced imaging modalities that were used in the studies perfusion‐based selection vs mismatch between diffusion‐weighted MRI (DWI) and fluid‐attenuated inversion recovery (FLAIR) MRI (DWI‐FLAIR mismatch), and (3) Utilization of mechanical thrombectomy in addition to IVT (IVT vs MT + IVT).

## Results

### Search and screening results

**Figure 1:**
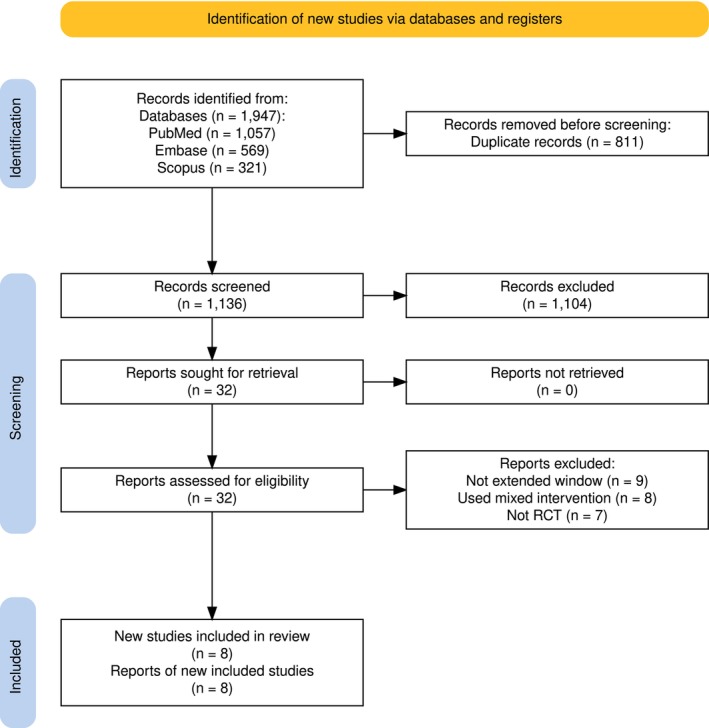
PRISMA flowchart detailing the literature review process.

The search retrieved 1947 records of which 811 duplicates were excluded and 1136 records were passed for further screening. Through the title and abstract screening stage, 1104 records were excluded, and 32 records were retained for full‐text screening. Finally, eight studies were determined to satisfy the inclusion criteria with the appropriate report of the outcomes of interest^3^, ^4^, ^7^, ^8^, ^9^, ^10^, ^11^, ^12^ (Fig. 1).

### Study characteristics

The included studies were eight clinical trials evaluating different thrombolytic agents in the ETW >4.5 h. ALT and TNK were used in four, and four studies, respectively. In all four studies of TNK, the dose of 0.25 mg/kg and a maximum dose of 25 mg were used. Three of the four ALT studies used standard dose of 0.9 mg/kg, and one study used 0.6 mg/kg.^7^ In two studies, the median time from LKW to treatment fell within the range of 4.5 to 9 h, while in another six studies, it exceeded 9 h. Males comprised 59% of cases. The mean age of patients ranged between 63 to 75 years among different studies. Different imaging modalities were used to assess viable brain tissue before treatment (patients selection), including DWI‐FLAIR mismatch in three studies, CT perfusion or perfusion–diffusion MRI (perfusion‐based selection) in four studies; and noncontrast CT scan in one study. The summary of study characteristics is presented in Table 1.

**Table 1:** Baseline study characteristics.

Author – year	Study	Type of thrombolytic	Male, *n*/*N* (%)	Age	Baseline NIHSS	Baseline ASPECT	Time to Needle	MT Performed	Hypertension, *n*/*N* (%)	Diabetes Mellitus, *n*/*N* (%)	Hyperlipidemia, *n*/*N* (%)	Atrial Fibrillation, *n*/*N* (%)	Definition of sICH
Koga 2020	THAWS – RCT	Alteplase	ALT: 45/70 (64) Control: 30/61 (49)	Mean age (SD) ALT: 73.2 ± 12.4 Control: 75.8 ± 11.9	Median NIHSS score (IQR) ALT: 7 (4–13) Control: 7 (5–12)	Median ASPECT score ALT: 9 (8–10) Control: 9 (8–10)	LKW to randomization – hour ALT: 10.2 (8.2–12.2) Control: 10.3 (7.7–11.8)	0	ALT: 49/70 (70) Control: 41/61 (67)	ALT: 14/70 (20) Control: 12/61 (20)	ALT: 23/70 (33) Control: 23/61 (38)	ALT: 27/70 (39) Control: 21/61 (34)	SITS‐MOST^a^
Ma 2019	EXTEND – RCT	Alteplase	ALT: 59/113 (52) Control: 66/112 (59)	Mean age (SD) ALT: 73.7 ± 11.7 Control: 71.0 ± 12.7	Median NIHSS score (IQR) TNK: 12.0 (8.0–17.0) Control: 10.0 (6.0–16.5)	NR	Onset to IVT – minute ALT: 432 (374–488) Control: 450 (374–500)	0	ALT: 84/113 (74) Control: 73/112 (65)	ALT: 26 (23) Control: 21 (19)	NR	NR	SITS‐MOST
Ringleb 2019	ECASS‐4 – RCT	Alteplase	ALT: 36/61 (59) Control: 31/58 (53)	Median age (IQR) – years ALT: 76 (65–83) Control: 79 (67–84)	Median NIHSS score (IQR) ALT: 10 (9) Control: 9 (10)	NR	Mid‐point between sleep onset and time of waking median (IQR) – hour ALT: 7.7 (1.5) Control: 7.3 (1.9)	NR	ALT: 42/61 (68.9) Control: 42/58 (72.4)	ALT: 16/61 (26.2) Control: 12/58 (20.7)	ALT: 31/61 (50.8) Control: 25/58 (41.4)	ALT: 14/61 (23) Control: 16/58 (27.6)	ECASS‐3^b^
Thomalla 2018	WAKE UP – RCT	Alteplase	ALT: 165/254 (65) Control: 160/249 (64)	Mean age (SD) ALT: 65.3 ± 11.2 Control: 65.2 ± 11.9	Median NIHSS score (IQR) ALT: 6 (4–9) Control: 6 (4–9)	NR	LKW to treatment median (IQR) – hour ALT: 10.3 (8.1–12.0) Control: 10.4 (8.1–12.1)	NR	ALT: 135/254 (53.1) Control: 131/249 (52.6)	ALT: 43/254 (16.9) Control: 39/249 (15.7)	ALT: 93/254 (36.6) Control: 85/249 (34.1)	ALT: 30/254 (11.8) Control: 29/249 (11.6)	ECASS‐3
Roaldsen 2023	TWIST – RCT	Tenecteplase	TNK: 164/288 (57) Control: 107/290 (37)	Median age (IQR) – years TNK: 73·9 (66·4–80·8) Control: 73·3 (65·8–82·0)	Median NIHSS score (IQR) TNK: 6 (5–11) Control: 6 (5–10)	Median ASPECT score TNK: 10 (10–10) Control: 10 (9–10)	LKW to randomization median (IQR) – minute TNK: 652 (553–774) Control: 653 (524–755)	TNK: 18/288 Control: 42/290	TNK: 176/276 (64) Control: 177/279 (63)	TNK: 55/278 (20) Control: 52/281 (19)	NR	TNK: 55/267 (21) Control: 31/272 (11)	SITS‐MOST
Wang 2023	ROSE‐TNK – RCT	Tenecteplase	TNK: 31/40 (77.5) Control: 26/40 (65)	Mean age (SD) TNK: 62.68 ± 8.87 Control: 62.80 ± 8.56	Median NIHSS score (IQR) TNK: 7.5 (6–10.75) Control: 7 (6–8.75)	NR	Onset to randomization Mean ± SD – hours TNK: 10.97 ± 4.67 hours Control:11.01 ± 4.14 hours	0	TNK: 24/40 (60) Control: 28/40 (70)	TNK: 9/40 (22.5) Control: 13/40 (32.5)	NR	NR	ECASS‐3
Albers 2024	TIMELESS‐RCT	Tenecteplase	TNK: 106/228 Control: 107/230	Median age (IQR) – years TNK: 72 (62–79) Control: 73 (63–82)	Median NIHSS score (IQR) TNK: 12 (8–17) Control: 12 (8–18)	Median ASPECT score TNK: 8.0 (6.0–8.0) Control: 8.0 (7.0–9.0)	Median duration (IQR) LKW to drug administration: TNK: 12.7 h (9.2–15.8) Control: 13.0 h (9.0–16.9)	TNK: 176/228 Control: 178/230	NR	NR	NR	NR	ECASS‐3
Xiong 2024	TRACE‐III – RCT	Tenecteplase	TNK:183/264 (69) Control: 167/252 (66)	Median age (IQR) – years TNK: 67 (58–75) Control: 68 (59–76)	Median (range) NIHSS score TNK: 11 (7–15) Control: 10 (7–14)	NR	LKW to randomization(IQR) 12.3 (8.5–16.4)	4 patients in the tenecteplase group and in 5 patients in Control	TNK: 177/264 (67.0) Control: 180/252 (71.4)	TNK: 69/264 (26.1) Control: 71/252 (28.2)	TNK: 81/264 (30.7) Control: 87/252 (34.5)	TNK: 49/ 264(18.6) Control: 48/252 (19.0)	ECASS‐3

ALT, alteplase; ASPECTS, Alberta stroke programme early CT; ECASS, European Cooperative Acute Stroke Study; LKW, last known well; NIHSS, The National Institutes of Health Stroke Scale or NIH Stroke Scale; NR, not reported; sICH, Symptomatic Intracranial hemorrhage; SITS‐MOST, Safe Implementation of Thrombolysis in Stroke Monitoring Study; TNK, Tenectaplase.

^a^ Definition of sICH based on SITS‐MOST included local or remote type 2 parenchymal hematoma on the imaging scan taken 22 to 36 h after treatment, along with neurological decline, indicated by an NIHSS score increase of 4 points or more from the baseline or the lowest value between baseline and 24 h, hemorrhage result in death.

^b^ Definition of sICH based on ECASS‐3 included any hemorrhage with neurologic deterioration, as indicated by an NIHSS score that was higher by 4 points or more than the value at baseline or the lowest value in the first 7 days, or any hemorrhage leading to death plus the hemorrhage must have been identified as the predominant cause of the neurologic deterioration.

### Risk of bias assessment

Risk of bias was assessed using RoB 2 tool. There was an overall low risk of bias in three studies, moderate risk of bias in four studies, and high risk of bias in one study (Table S1).

### Functional outcomes

**Figure 2:**
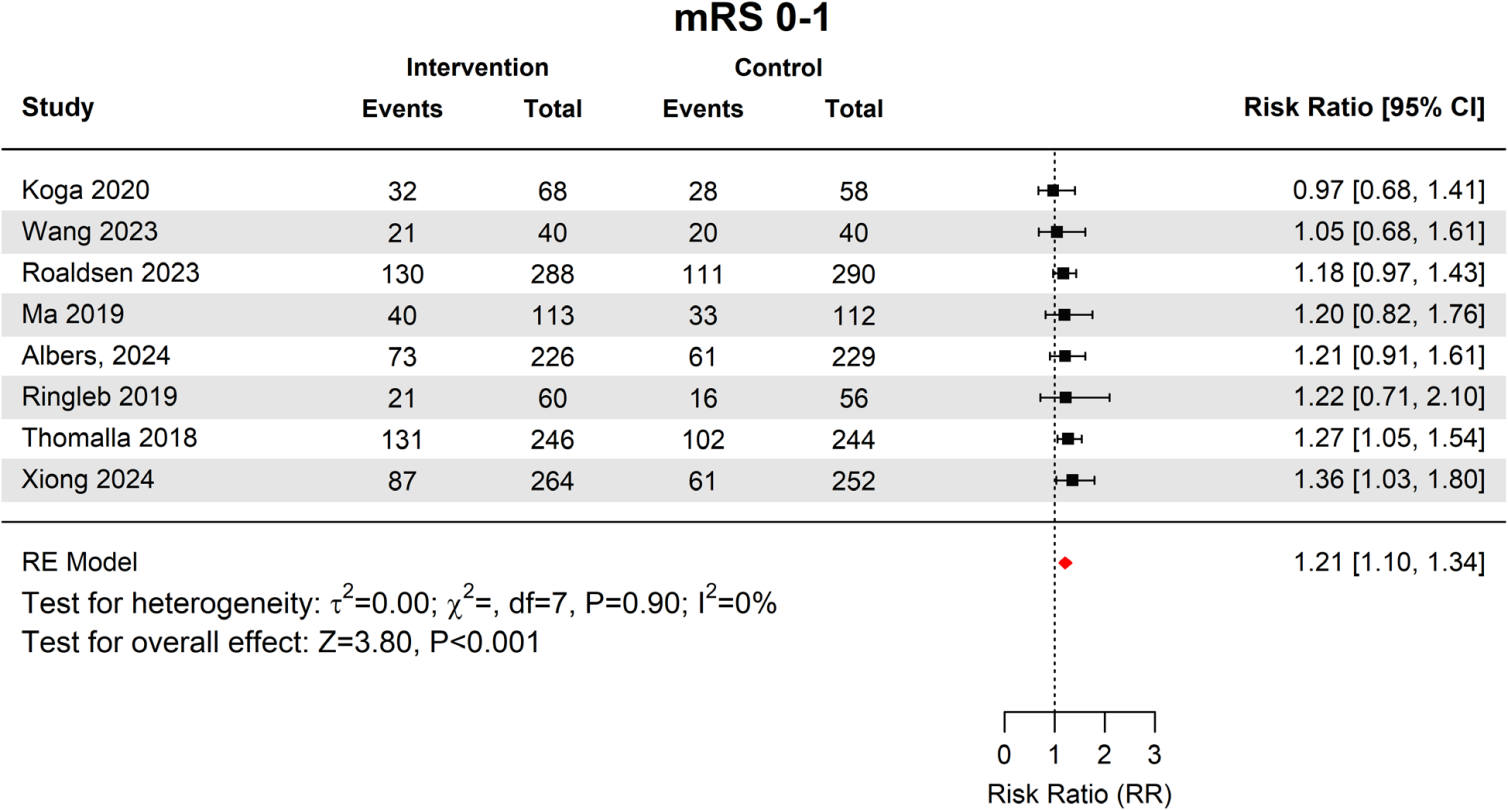
Forest plot of favorable functional outcomes defined as mRS 0–1.

In the intervention group, 535 out of 1305 patients, 41.1% (95% CI [35.3–47]) achieved an excellent outcome (mRS score 0–1), compared to 432 out of 1281 patients, 34.6% (95% CI [28.7–41]) in the control group (RR 1.21, 95% CI 1.1–1.34, *p* < 0.001) (Fig. 2). There was no heterogeneity across studies (*I* ^2^ = 0%, *p* = 0.9).

**Figure 3:**
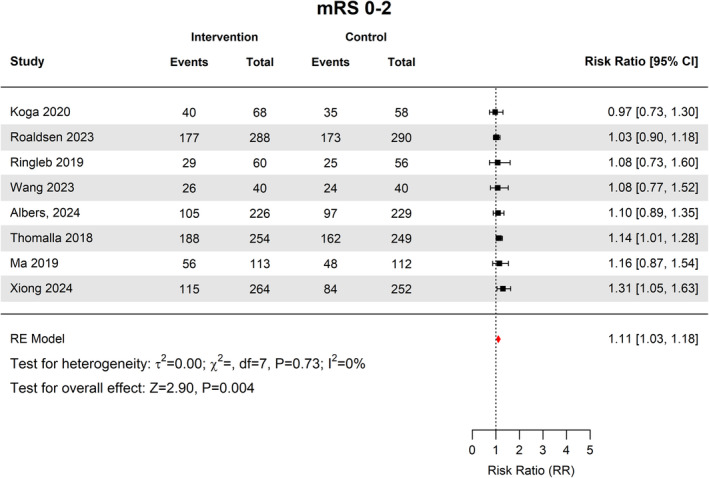
Forest plot of good functional outcomes defined as mRS 0–2.

Additionally, 736 out of 1313 patients, 56.1% (95% CI [48.3–63.5]) in the intervention group had good functional outcomes (mRS 0–2), versus 648 out of 1286 patients, 49.2% (95% CI [40.5–58]) in the control group (RR 1.11, 95% CI 1.03–1.18, *p* = 0.004) (Fig. 3). There was no heterogeneity across studies (*I* ^2^ = 0%, *p* = 0.7).

Subgroup analysis by type of thrombolytic agent for the endpoint mRS 0–1 demonstrated that both ALT (*p* = 0.012) and TNK (*p* = 0.004) lead to a higher rate of excellent outcomes compared to controls when administered in ETW (Fig. S1).

For the endpoint mRS 0–2, treatment with ALT was significantly better than controls (*p* = 0.02). A marginally significant trend was also observed for TNK (RR 1.10 95% CI 1.0–1.21, *p* = 0.059) (Fig. S2).

The subgroup analysis based on imaging modality showed statistically significant higher excellent functional recovery between IVT group and controls in studies that used a combination of CT perfusion and perfusion–diffusion MRI (perfusion imaging) (*p* = 0.006). However, studies that used DWI‐FLAIR mismatch did not show a better excellent functional recovery following IVT treatment in an extended window (*p* = 0.11) (Fig. S3). In addition, both imaging selection groups (perfusion imaging and DWI‐FLAIR mismatch) showed good functional recovery (mRS 0–2) with IVT use in ETW (Fig. S4).

Both the MT + IVT and IVT alone groups demonstrated excellent functional recovery, as shown in the mRS 0–1 (Fig. S5).

### Symptomatic intracranial hemorrhage

**Figure 4:**
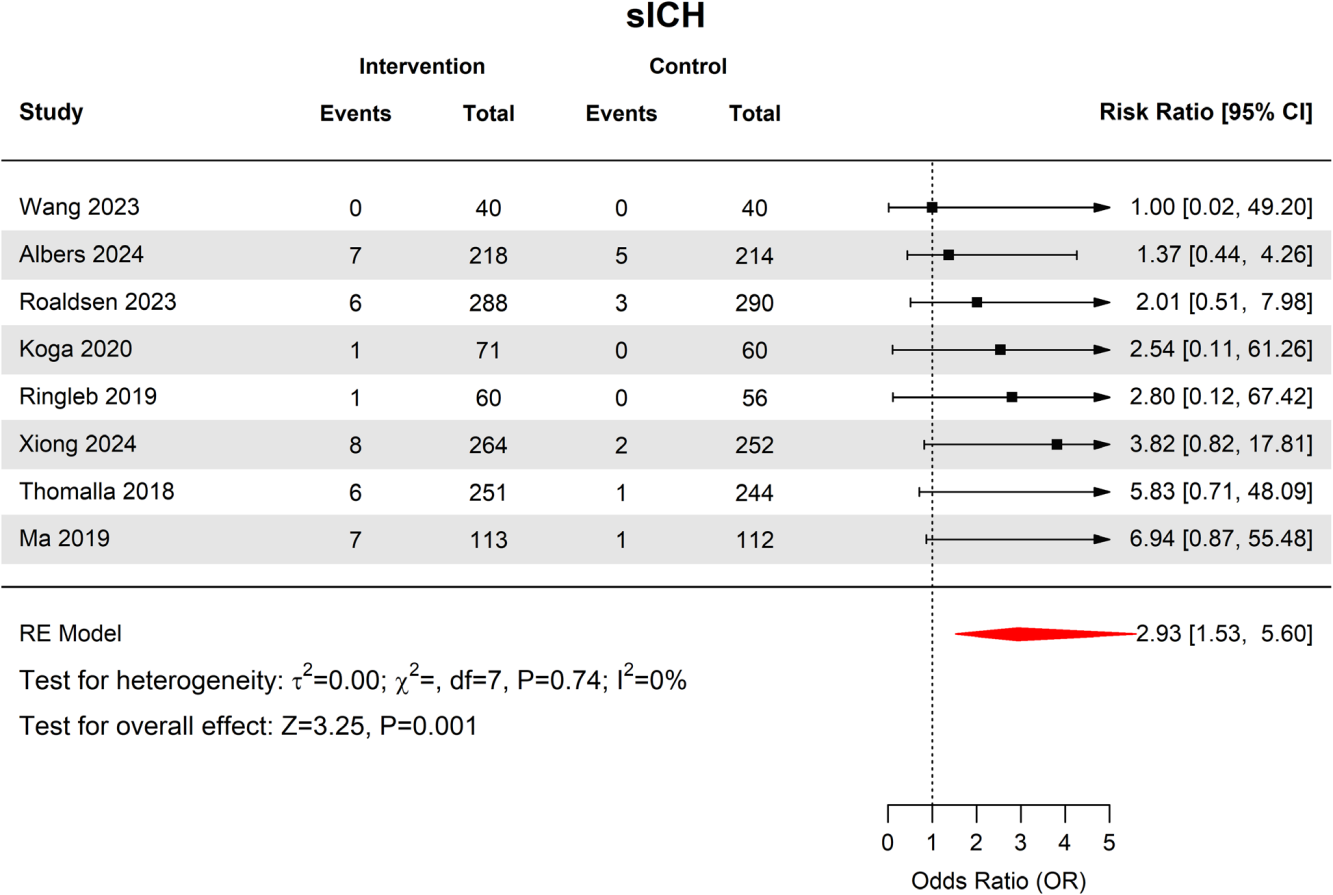
Forest plot of rates of sICH.

Overall, sICH was observed in 36 patients from 1305 patients in the thrombolytic group (2.9%, 95% CI [2.1–4.2]) compared with 8 from 1268 patients in the control group (0.8%, 95% CI [0.3–2.1]). This difference was statistically significant (RR 2.11 95% CI 1.30–3.43, *p* = 0.003) (Fig. 4). There was no heterogeneity among studies (*I* ^2^ = 0%, *p* = 0.68).

Of note, subgroup analysis by thrombolytic agent showed a significant increased risk of sICH for ALT (RR 7.28 95% CI 1.68–31.6, *p* = 0.008) and an insignificant risk of sICH for TNK (RR 2.06 95% CI 0.98–4.33, *p* = 0.058) (Fig. S7).

Risk of sICH was elevated regardless of method of imaging patients selection (*p* = 0.016) for DWI‐FLAIR mismatch and (*p* = 0.008) for perfusion‐based patient selection (Fig. S8).

Studies that contained patients who underwent MT (MT + IVT) did not show higher risk of sICH (*p* = 0.078) (Fig. S9). However, risk of sICH was higher if only IVT was used (*p* = 0.016).

### Mortality

**Figure 5:**
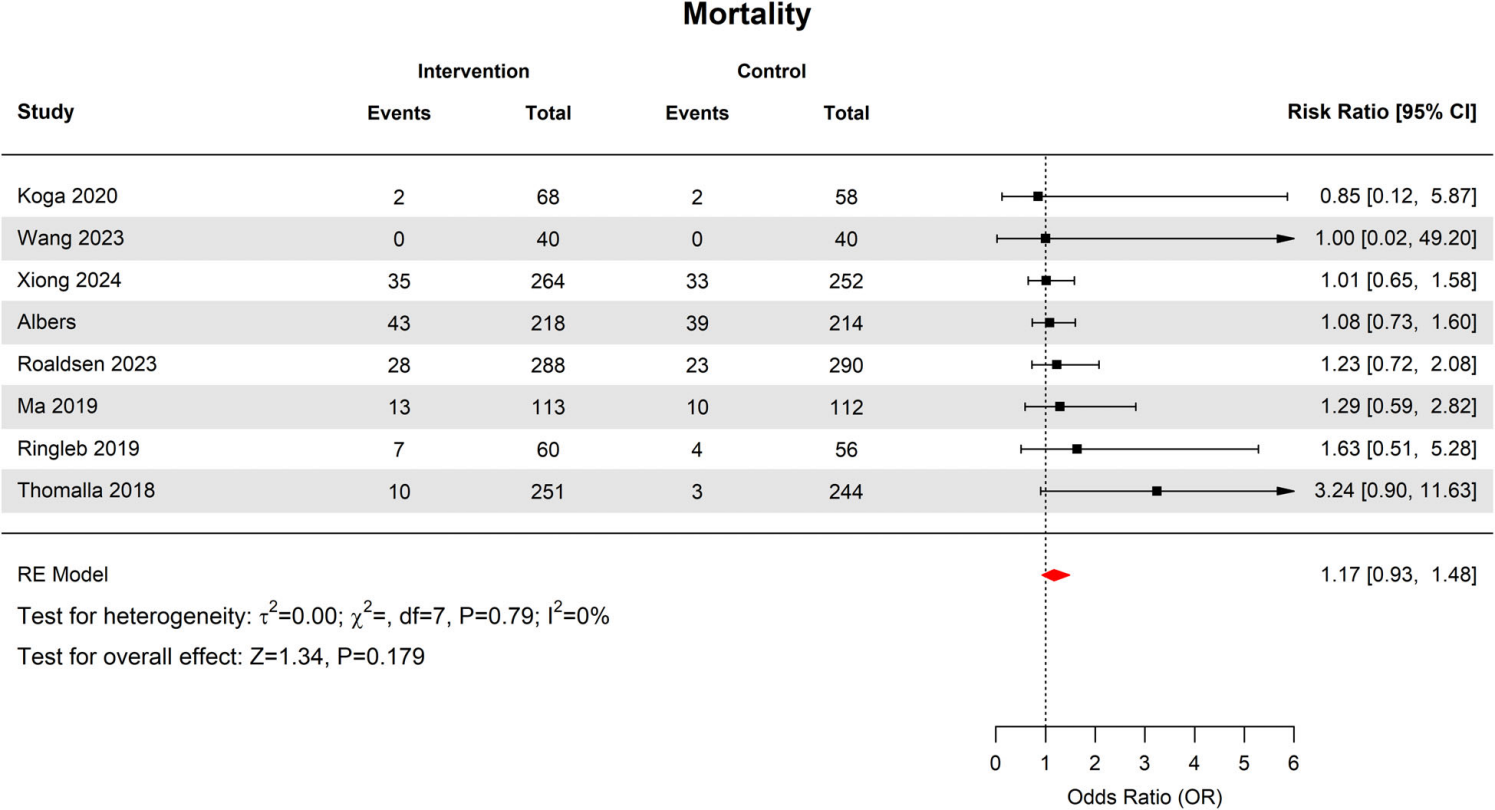
Forest plot of mortality rates.

Overall, mortality was observed in 138 of 1302 patients in the thrombolytic group (8.3%, 95% CI [5–13.6]) compared with 114 of 1266 patients in the control group (6.0%, 95% CI [3–11.6]). This difference was not statistically significant (RR 1.17 95% CI 0.93–1.48, *p* = 0.17) (Fig. 5). There was no heterogeneity across studies (*I* ^2^ = 0%, *p* = 0.79). Subgroup analyses by thrombolytic agent, imaging modality and in those who received MT with IVT showed no difference in rate of mortality among subgroups (Figs. S10–S12).

## Discussion

This systematic review and meta‐analysis included 8 randomized clinical trials with a combined total of 2221 patients. Our results show that patients treated with IV thrombolysis within the ETW of 4.5 up to 24 h since LKW had higher rates of favorable outcomes than those who did not receive thrombolytic therapy. These favorable outcomes were observed despite an increase in the rate of sICH among patients treated with thrombolysis in the ETW, and there was no difference in mortality.

The meta‐analysis showed beneficial effects of IV thrombolysis when evaluating excellent functional outcome (mRS 0–1) and absence of disability (mRS 0–2) at 90 days. As expected, risk of sICH was increased after thrombolysis after 4.5 h. However, the degree of increased sICH is not out of proportion to what has been observed within the first 4.5 h^13^ and the higher risk of hemorrhage did not translate into a greater risk of mortality. Of note, the rate of sICH is significant in those who received ALT as opposed to the insignificant rates of sICH in those who were treated with TNK which is consistent with the results from CERTAIN trial.^14^ This is likely due to the unique pharmaceutical differences between the ALT and TNK in that TNK has higher affinity to fibrin and theoretically is associated with lower risks of hemorrhage.^15^, ^16^ Notably, a recent meta‐analysis evaluating the safety and efficacy of TNK for AIS in the ETW found that TNK is both efficacious and has a favorable safety profile based on data from three included RCTs.^17^ The efficacy finding from the latter study is consistent with this current meta‐analysis.

Criteria for imaging selection varied across studies, with some using perfusion scans or MRI scans to identify salvageable brain tissue and others relying on noncontrast CT. Of note, higher rates of patients achieved excellent functional outcomes were noted in studies that used perfusion imaging to select patients for IVT in the ETW >4.5 h as opposed to studies that used DWI‐FLAIR mismatch which is consistent with previous literature report that stated using perfusion imaging to select patients for IVT improves prognostication and identification of patients for IVT beyond the conventional therapeutic window.^18^

Our meta‐analysis provides a comprehensive analysis of the outcomes of IVT in the ETW and includes subgroup analyses of the safety and efficacy outcomes by thrombolytic agent, selection imaging modalities as well as patient treated with MT in addition to IVT. However, it has several limitations. Some of the studies included had small sample sizes, and other studies halted the investigation without reaching the expected patient enrollment. We were not able to adjust for stroke severity at onset. Imaging criteria for inclusion and definitions of sICH differed across studies. Subgroup analyses were limited by varying statistical power.

Overall, the results of this meta‐analysis indicate that IV thrombolysis may be effective to improve functional outcomes for selected patients treated beyond the currently recommended therapeutic window of 4.5 h since LKW, despite an increased risk of sICH. The optimal criteria for selection, thrombolytic agent, and longest time to treatment from LKW remain to be determined by future investigations.

## Conclusion

Use of IVT in the extended window is beneficial especially with the use of advance imaging for patients' selection.

## Funding Information

None.

## Conflict of Interest

DFK holds equity in Nested Knowledge, Superior Medical Editors, and Conway Medical, Marblehead Medical and Piraeus Medical. He receives grant support from MicroVention, Medtronic, Balt, and Insera Therapeutics; has served on the Data Safety Monitoring Board for Vesalio; and received royalties from Medtronic.

## Author Contributions

OMA, YMM, and SBJ contributed to the conception and design of the study; OMA, SBJ, and MAT contributed to the acquisition and analysis of data; OMA, SBJ, YMM, SY, AAR, and DFK contributed to drafting the text or preparing the figures. OMA and SBJ contributed equally to this manuscript.

## Informed Consent

Informed consent was not required for this study.

## Supporting information


Data S1.

Appendix S1.

## Data Availability

The data that support the findings of this study were derived from the following references [3, 4, 7, 8, 9, 10, 11, 12].
