# Supplementary material for: Safety and efficacy of intravenous thrombolytic therapy in the extended window up to 24 hours: A systematic review and meta‐analysis
Source: Ann Clin Transl Neurol. 2024 Oct 29;11(12):3310–9. doi: 10.1002/acn3.52239 (PMC11651193; doi:10.1002/acn3.52239)

Search strategy for Embase:

(‘stroke’/exp OR ‘stroke’ OR ‘acute ischemic stroke’/exp OR ‘acute ischemic stroke’ OR ‘ais’)AND (‘tissue plasminogen activator’ OR ‘alteplase’ OR ‘rtpa’ OR ‘tenecteplase’/exp OR ‘tenecteplase’ OR ‘tnk-tpa’/exp OR ‘tnk-tpa’ OR ‘tnk’/exp’ OR ‘tnk’ OR thrombolytic therapy’ OR ‘intravenous thrombolysis’ OR ‘intravenous alteplase’ OR ‘ivt’ OR desmoteplase’ OR ‘dspa’) AND (‘beyond 4.5 hours after stroke’ OR > ‘4.5 hours after stroke’ OR ‘extended therapeutic window’ OR ‘4.5 hours to 24 hours’ OR ‘wake-up’ OR ‘unknown last know well’ OR ‘unknown onset’)

Supplementary table 1 Risk of bias assessment of studies using RoB 2 assessment tool.

Unique ID	D1	D2	D3	D4	D5	Overall
Roaldsen, 2023	+	+	+	+	+	+
Wang, 2023	+	!	+	+	+	!
Koga, 2020	!	!	+	+	+	!
Ma, 2019	-	!	+	+	+	-
Ringleb, 2019	+	+	!	+	+	!
Thomalla, 2018	+	+	+	+	+	+
Xiong, 2024	!	+	+	+	+	!
Albers, 2024	+	+	+	+	+	+
D1: Selection of the reported result, D2: Measurement of the outcome, D3: Missing outcome data, D4: Deviations from intended intervention, D5: Randomization process						

Supplementary Figure 1. Forest Plot for Excellent Functional Outcomes Based on Thrombolytic Agents.

mRS0_1 subgroup analysis based on thrombolytic agent

Study	Intervention Events	Intervention Total	Control Events	Control Total	Risk Ratio [95% CI]
**Alteplase**					
Ringleb 2019	21	60	16	56	1.22 [0.71, 2.10]
Thomalla 2018	131	246	102	244	1.27 [1.05, 1.54]
Koga 2020	32	68	28	58	0.97 [0.68, 1.41]
Ma 2019	40	113	33	112	1.20 [0.82, 1.76]
RE Model for Subgroup					1.21 [1.04, 1.40]
Test for overall subgroup effect: Z=2.50, P=0.012					
**Tenecteplase**					
Albers, 2024	73	226	61	229	1.21 [0.91, 1.61]
Xiong 2024	87	264	61	252	1.36 [1.03, 1.80]
Wang 2023	21	40	20	40	1.05 [0.68, 1.61]
Roaldsen 2023	130	288	111	290	1.18 [0.97, 1.43]
RE Model for Subgroup					1.21 [1.06, 1.38]
Test for overall subgroup effect: Z=2.86, P=0.004					

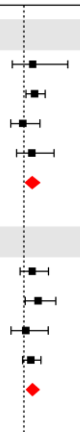

Supplementary Figure 2. Forest Plot for Good Functional Outcomes Based on Thrombolytic Agents.

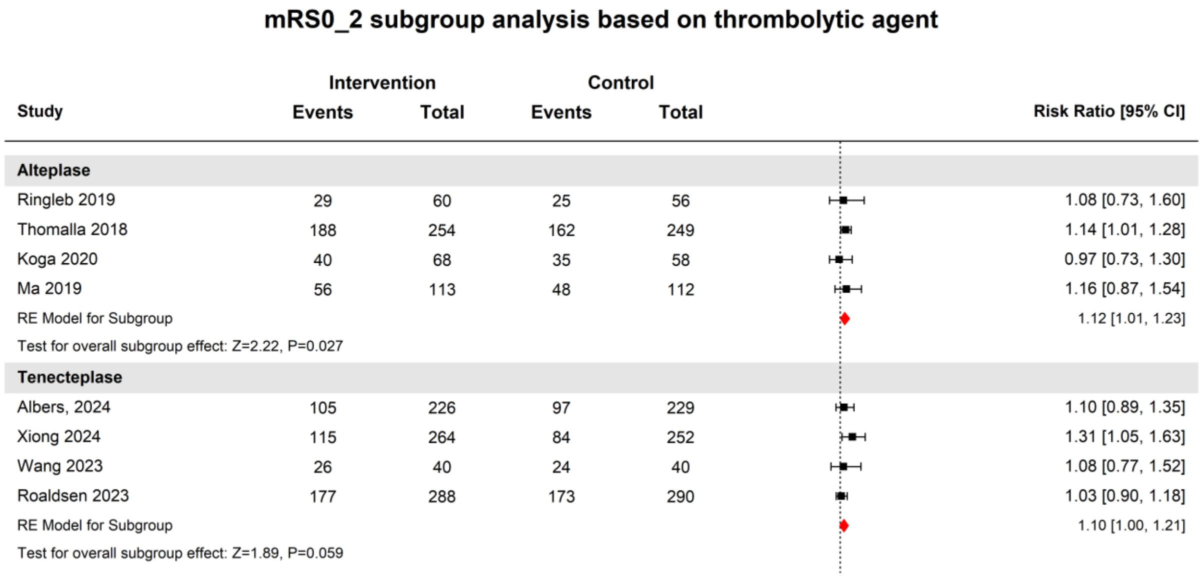

Supplementary Figure 3. Forest Plot for Excellent Functional Outcomes Based on Imaging Modality for Patient's Selection.

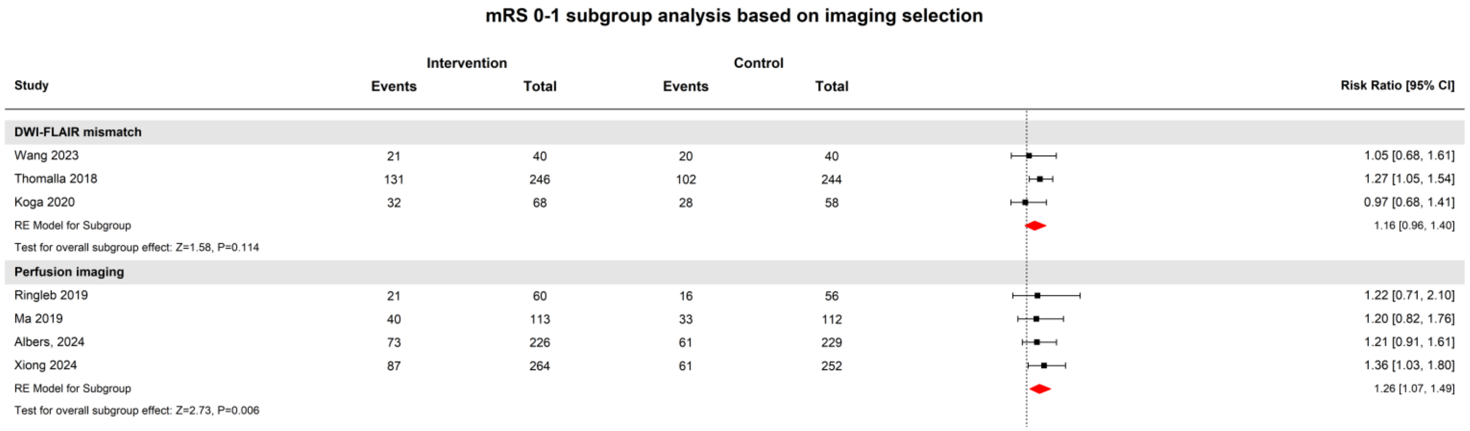

Supplementary Figure 4. Forest Plot for Good Functional Outcomes Based on Imaging Modality for Patient's Selection.

mRS 0-2 subgroup analysis based on imaging selection

Study	Intervention Events	Intervention Total	Control Events	Control Total	Risk Ratio [95% CI]
**DWI-FLAIR mismatch**					
Wang 2023	26	40	24	40	1.08 [0.77, 1.52]
Thomalla 2018	188	254	162	249	1.14 [1.01, 1.28]
Koga 2020	40	68	35	58	0.97 [0.73, 1.30]
RE Model for Subgroup					1.11 [1.00, 1.23]
Test for overall subgroup effect: Z=1.99, P=0.046					
**Perfusion imaging**					
Ma 2019	56	113	48	112	1.16 [0.87, 1.54]
Albers, 2024	105	226	97	229	1.10 [0.89, 1.35]
Xiong 2024	115	264	84	252	1.31 [1.05, 1.63]
Ringleb 2019	29	60	25	56	1.08 [0.73, 1.60]
RE Model for Subgroup					1.17 [1.03, 1.33]
Test for overall subgroup effect: Z=2.46, P=0.014					

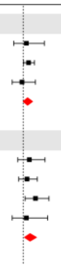

Supplementary Figure 5. Forest Plot for Excellent Functional Outcomes Based on Combined IVT and MT.

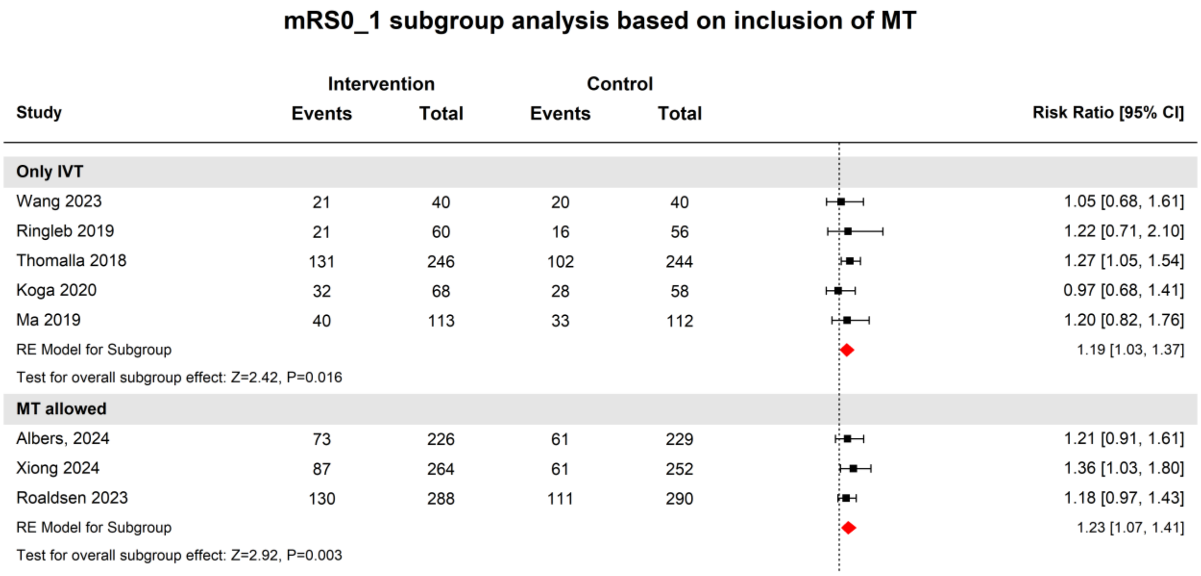

Supplementary Figure 6. Forest Plot for Good Functional Outcomes Based on Combined IVT and MT.

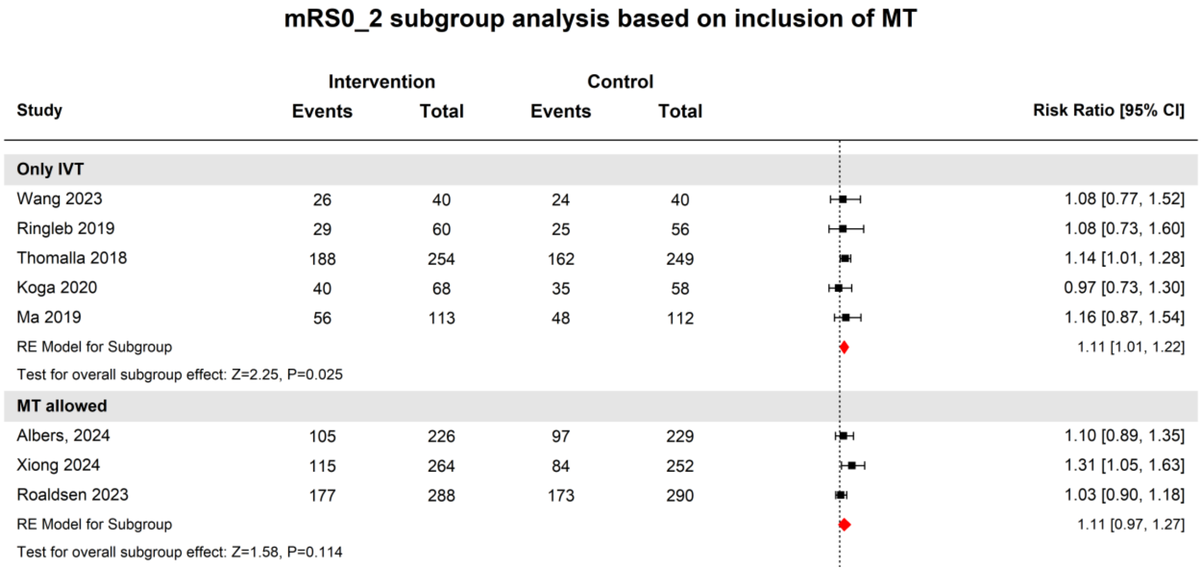

Supplementary Figure 7. Forest Plot for Rates of sICH Based on Thrombolytic Agents.

sICH subgroup analysis based on thrombolytic agent

Study	Intervention Events	Intervention Total	Control Events	Control Total	Risk Ratio [95% CI]
**Alteplase**					
Ringleb 2019	1	60	0	56	2.80 [0.12, 67.42]
Thomalla 2018	20	251	12	244	1.62 [0.81, 3.24]
Koga 2020	1	71	0	60	2.54 [0.11, 61.26]
Ma 2019	7	113	1	112	6.94 [0.87, 55.48]
RE Model for Subgroup					2.15 [1.14, 4.07]
Test for overall subgroup effect: Z=2.35, P=0.019					
**Tenecteplase**					
Albers 2024	7	218	5	214	1.37 [0.44, 4.26]
Xiong 2024	8	264	2	252	3.82 [0.82, 17.81]
Wang 2023	0	40	0	40	1.00 [0.02, 49.20]
Roaldsen 2023	6	288	3	290	2.01 [0.51, 7.98]
RE Model for Subgroup					2.06 [0.97, 4.33]
Test for overall subgroup effect: Z=1.89, P=0.058					

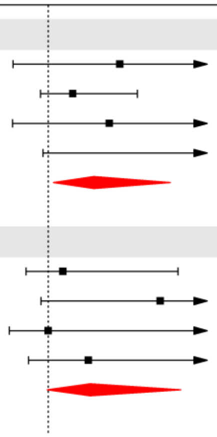

Supplementary Figure 8. Forest Plot for Rates of sICH Based on Imaging Modality for Patient's Selection.

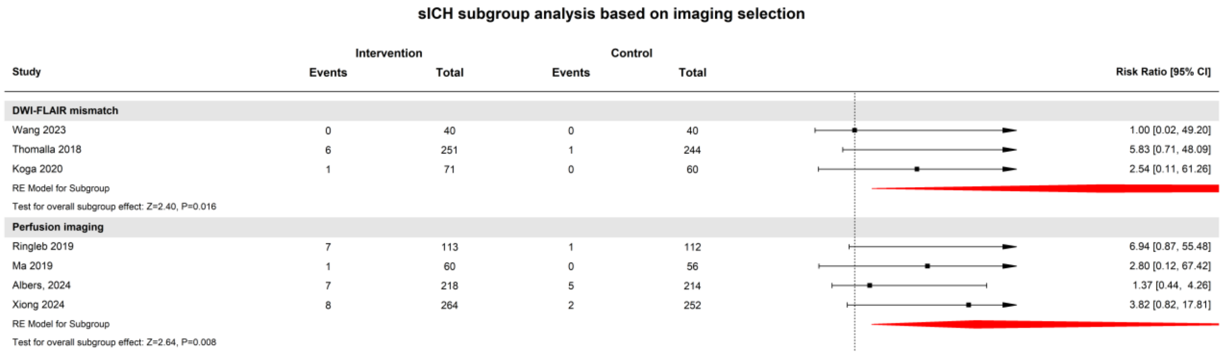

Supplementary Figure 9. Forest Plot for Rates of sICH Based on Combined IVT and MT.

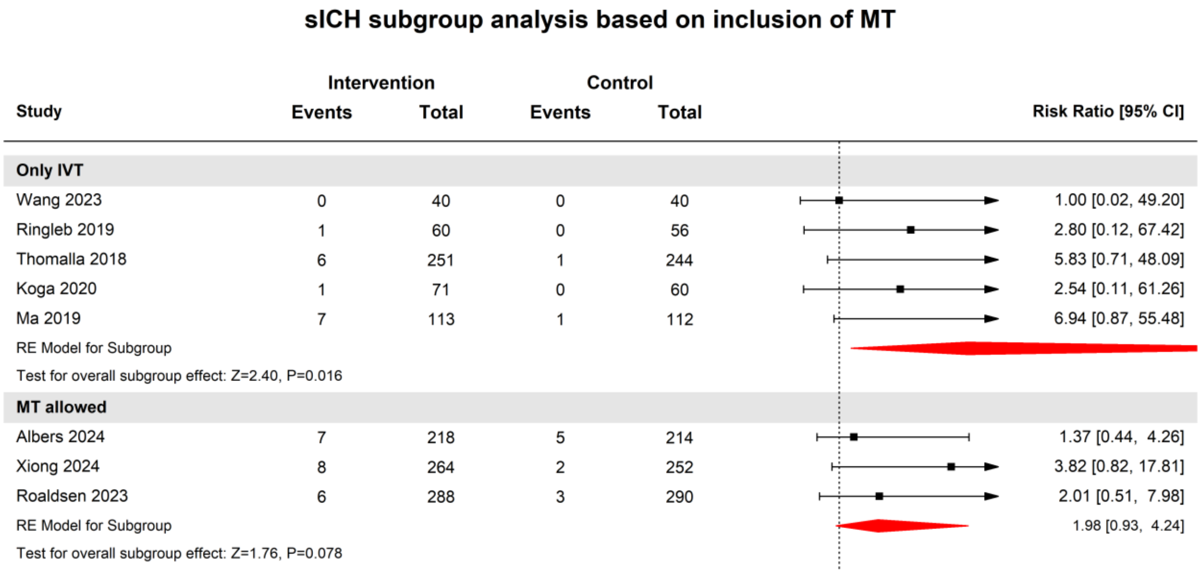

Supplementary Figure 10. Forest Plot for Mortality Rates Based on Thrombolytic Agents.

Mortality subgroup analysis based on thrombolytic agent

Study	Intervention Events	Intervention Total	Control Events	Control Total	Risk Ratio [95% CI]
**Alteplase**					
Ringleb 2019	7	60	4	56	1.63 [0.51, 5.28]
Thomalla 2018	10	251	3	244	3.24 [0.90, 11.63]
Koga 2020	2	68	2	58	0.85 [0.12, 5.87]
Ma 2019	13	113	10	112	1.29 [0.59, 2.82]
RE Model for Subgroup					1.61 [0.93, 2.78]
Test for overall subgroup effect: Z=1.70, P=0.089					
**Tenecteplase**					
Albers	43	218	39	214	1.08 [0.73, 1.60]
Xiong 2024	35	264	33	252	1.01 [0.65, 1.58]
Wang 2023	0	40	0	40	1.00 [0.02, 49.20]
Roaldsen 2023	28	288	23	290	1.23 [0.72, 2.08]
RE Model for Subgroup					1.09 [0.84, 1.41]
Test for overall subgroup effect: Z=0.66, P=0.508					

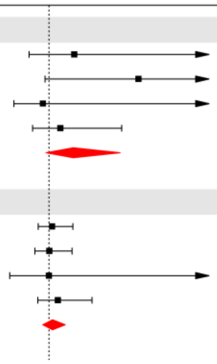

Supplementary Figure 11. Forest Plot for Mortality Rates Based on Imaging Modality for Patient's Selection.

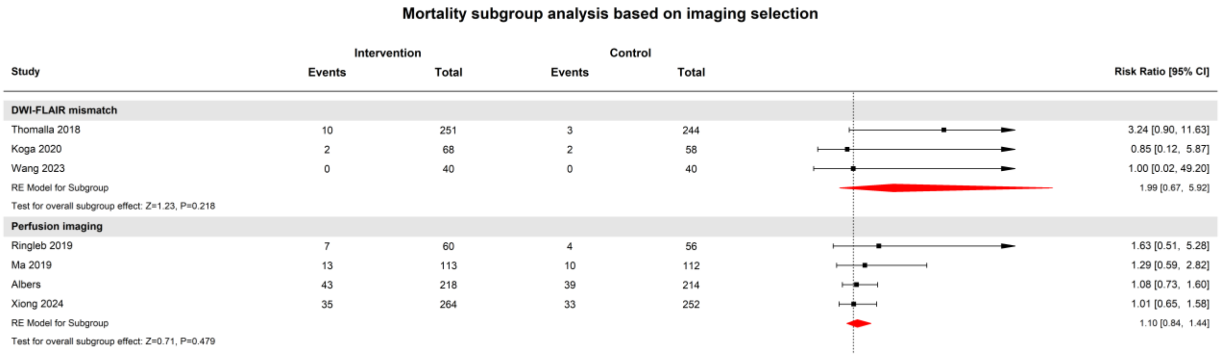

Supplementary Figure 12. Forest Plot for Mortality Rates Based on Combined IVT and MT.

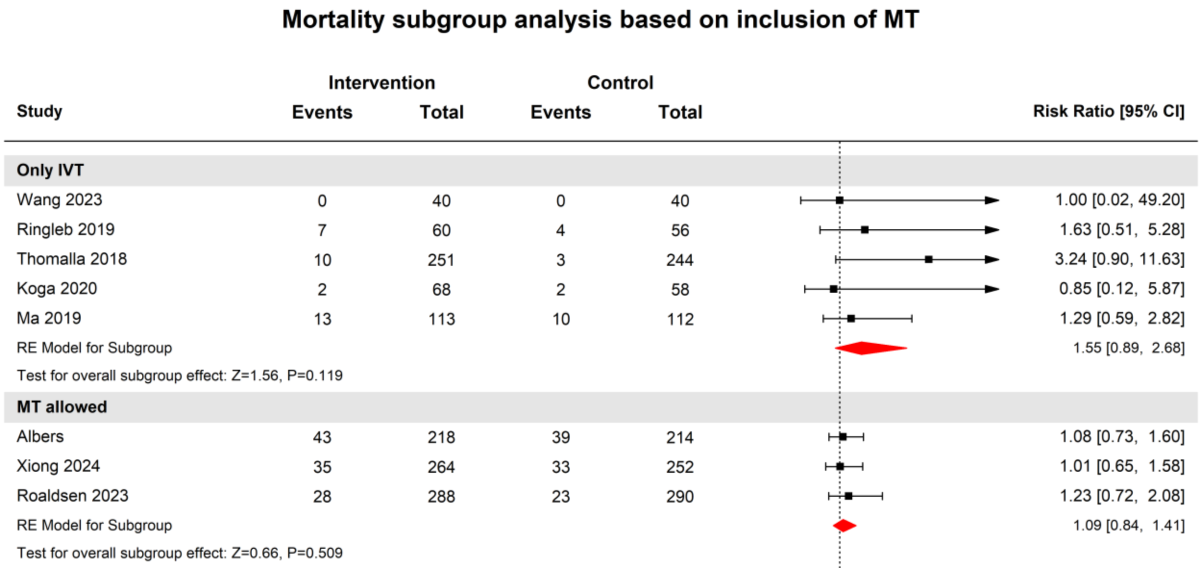

Supplement: Supplementary file 2 — Appendix S1. [file ACN3-11-3310-s001.pdf]
